# Orthogonal prediction of counterfactual outcomes

**DOI:** 10.1515/jci-2024-0051

**Published:** 2025-11-27

**Authors:** Stijn Vansteelandt, Paweł Morzywołek

**Affiliations:** 26656 Ghent University, Ghent, Belgium; University of Washington, Seattle, WA, USA

**Keywords:** causal prediction, DR-learner, heterogeneous treatment effect, meta-learner, orthogonal learner, R-learner, 62G08

## Abstract

Orthogonal meta-learners, such as DR-learner (Kennedy EH. Towards optimal doubly robust estimation of heterogeneous causal effects. arXiv preprint arXiv:2004.14497 2020), R-learner (Nie X, Wager S. Quasi-oracle estimation of heterogeneous treatment effects. Biometrika 2021;108:299–319) and IF-learner (Curth A, Alaa AM, van der Schaar M. Estimating structural target functions using machine learning and influence functions. arXiv preprint arXiv:2008.06461 2020), are increasingly used to estimate conditional average treatment effects. They are hoped to improve convergence rates relative to naïve meta-learners (e.g., T-, S- and X-learner (Künzel SR, Sekhon JS, Bickel PJ, Yu B. Metalearners for estimating heterogeneous treatment effects using machine learning. Proc Natl Acad Sci 2019;116:4156–65)) through de-biasing procedures that involve applying standard learners to specifically transformed outcome data. This leads them to disregard the possibly constrained outcome space, which can be particularly problematic for dichotomous outcomes: these typically get transformed to values that are no longer constrained to the unit interval, which may cause instability and makes it difficult for standard learners to guarantee predictions within the unit interval. To address this, we construct a non-orthogonal imputation-learner and an orthogonal ‘i-learner’ for the prediction of counterfactual outcomes, which respect the outcome space. These are more generally expected to outperform existing learners, even when the outcome is unconstrained, as we confirm empirically in simulation studies and an analysis of critical care data. Our development also sheds broader light onto the construction of orthogonal learners for other estimands.

## 1 Introduction

Data-adaptive modeling (e.g., based on model selection or machine learning algorithms) is routinely used by statisticians and data scientists to quantify associations and evaluate the effects of exposures, treatments or interventions. A well studied example concerns estimation of the mean of a counterfactual outcome *Y* ^1^, which represents the outcome that would be seen for a random subject if it were treated. Under standard causal assumptions, primarily that conditioning on a measured, possibly high-dimensional collection of variables *L* suffices to adjust for confounding of the effect of treatment *A*, coded 0 or 1, on outcome *Y*, this can be identified as [1]

$$E\left(Y^{1}\right)=E\left\{E\left(Y|A=1,L\right)\right\}.$$

Estimation may then proceed by first estimating the infinite-dimensional nuisance parameter *E*(*Y*|*A* = 1, *L*) using data-adaptive prediction algorithms, trained in the subsample of treated individuals, and next averaging these predictions over the entire sample (using a simple sample average). Such prediction algorithms – and more generally, nearly all model selection or machine learning algorithms – optimally balance bias versus variance in order to minimize expected (in-sample) prediction error. However, in doing so, they deliver no guarantees in terms of optimizing relevant performance measures (e.g., mean squared error) for the estimand of interest (e.g., for *E*(*Y* ^1^)). In fact, naïve use of data-adaptive strategies is well known to result in bias and excess variability [2], [3], [4], [5], [6]. This bias may be the result of eliminating variables that are strongly associated with the exposure of interest, thereby inducing confounding bias [7]; more generally, it is the result of oversmoothing in the wrong parts of the data (e.g., at the wrong covariate levels).

In recent years, enormous progress has been made in terms of making estimators of scalar estimands, like *E*(*Y* ^1^), less susceptible to the bias that affects the (possibly non-parametric) data-adaptive estimators (e.g., of *E*(*Y*|*A* = 1, *L*)) on which they are based. These developments almost exclusively rely on the so-called efficient influence curve or canonical gradient [8], 9] of the considered scalar estimands. They use it either to directly de-bias naïve estimators [6], or instead to base naïve estimators on data-adaptive estimators that are better targeted towards the parameter of interest [5]. The resulting theory is generally well developed, but limited to so-called pathwise differentiable parameters that are estimable at parametric (i.e. root-*n*) rates. It therefore does not readily extend to infinite-dimensional parameters, such as the conditional mean *E*(*Y* ^1^|*Z*) of a counterfactual outcome, with *Z* ⊆ *L*. The estimation of such quantities is nonetheless of increasing interest for personalized decision-making assisted by counterfactual outcome predictions [10], 11].

Foster and Syrgkanis [12] made progress in this infinite-dimensional setting by using so-called orthogonal learners; see ref. [13] for alternative developments. These are learners obtained by minimizing a so-called Neyman-orthogonal loss function. They do this by extending the key property of Neyman-orthogonality of influence curves to loss functions on which data-adaptive algorithms rely, with the aim to de-bias. Here, Neyman-orthogonality of a functional refers to the mean zero property of its directional derivatives [12] along one-dimensional paths that (only) change one of the nuisance parameters (e.g., *E*(*Y*|*A* = 1, *L*) or *P*(*A* = 1|*L*)) on which it is based, no matter which. A loss function is called Neyman-orthogonal when this property holds for all its directional derivatives along one-dimensional paths that change the (infinite-dimensional) parameter of interest. The theory of [12] is generic and, like [14], provides suggestions for how Neyman-orthogonal loss functions can be constructed. However, the resulting loss functions can be difficult to optimize using off-the-shelf machine learning algorithms, a problem that we will address in this paper.

Specific orthogonal learners for *E*(*Y* ^1^ − *Y* ^0^|*Z*) are given in [15], [16], [17], [18], with the first three learners readily extending to the estimation of *E*(*Y* ^1^|*Z*) (see later), and an additional such learner being proposed in ref. [12]. These learners share a number of limitations, which we aim to address here. First, it is not readily clear what these learners deliver when, because of smoothing, the loss function is optimized over a function class that does not contain the truth (see ref. [19] for further insight into this). Second, these learners have a key limitation in that they apply standard learners to transformed outcome data, which leads them to disregard the possibly constrained outcome space. For instance, a DR-learner for *E*(*Y* ^1^|*Z*) [15] would amount to regressing pseudo-outcomes

$$\frac{A}{P\left(A=1|L\right)}\left\{Y-E\left(Y|A=1,L\right)\right\}+E\left(Y|A=1,L\right)$$

onto *Z*, with nuisance parameters *P*(*A* = 1|*L*) and *E*(*Y*|*A* = 1, *L*) substituted by data-adaptive estimates. This is particularly problematic for dichotomous outcomes since these pseudo-outcomes are not constrained to the unit interval, causing instability and making it difficult for standard learners to guarantee predictions within the unit interval.

In this article we will remedy the first problem by finding the function *m*(*Z*) in some functional class Γ, e.g., the set of all (measurable) functions of *Z* with finite second moment, which minimises a de-biased estimator of the counterfactual prediction error

(1) $$E\left[{\left\{Y^{1}-m\left(Z\right)\right\}}^{2}\right].$$

A key challenge is that this de-biased estimator does not readily lend itself to minimization using off-the-shelf software for statistical/machine learning. Inspired by targeted learning algorithms [5], we therefore next target the infinite-dimensional nuisance parameters in such a way that this de-biased estimator of the loss reduces to a standard mean squared error loss w.r.t. imputed counterfactuals *Y* ^1^. In doing so, we address the second problem mentioned above. We show that the resulting learner is Neyman-orthogonal, and refer to it as i-learner or imputation-learner, in view of its reliance on imputed outcomes that are ‘orthogonalized’ or targeted towards the estimation of counterfactual (conditional) means. Simulation studies and an analysis of critical care data show adequate performance, even for unconstrained outcomes.

## 2 Problem

Consider a study design which collects independent and identically distributed (i.i.d.) data on a possibly high-dimensional vector of covariates *L*, that suffices to adjust for confounding of the effect of a dichotomous treatment *A* on an outcome *Y*, in the sense that *Y* ^1^ ⊥⊥ *A*|*L*. Suppose furthermore that the consistency assumption holds that *Y* ^1^ equals *Y* in distribution for individuals with *A* = 1, and that *P*(*A* = 1|*L*) > *σ* with probability 1 (w.p.1) for some *σ* > 0. Our aim is to find the function *m*(*Z*) in Γ which minimises the counterfactual prediction error (1). If Γ contains *E*(*Y* ^1^|*Z*), then the above minimization problem leads to *m*(*Z*) = *E*(*Y* ^1^|*Z*), but otherwise delivers the closest approximation (in mean squared error). When *Z* = *L*, *E*(*Y* ^1^|*Z*) reduces to *E*(*Y*|*A* = 1, *L*) under the stated identification assumptions. Often, however, we may choose *Z* to be a small subset of *L*, either because it is logistically better feasible in clinical practice to predict *Y* ^1^ based on a small collection of variables, or because the uncertainty in the resulting predictions can be more accurately expressed when *Z* is low-dimensional. In that case, the minimizer to the counterfactual prediction error can be identified as $E\left\{E\left(Y|A=1,L\right)|Z\right\}$ under the stated assumptions.

### 2.1 Minimizing counterfactual prediction error

The counterfactual prediction error (1) does not readily provide a feasible loss function for practical use. This is partly because of it being expressed in terms of population expectations, but more importantly because *Y* ^1^ is only measured for subjects with *A* = 1. This can be remedied by instead optimizing a consistent estimator of the counterfactual prediction error. For this, we may use inverse probability weighting upon noting that

$$E\left[{\left\{Y^{1}-m\left(Z\right)\right\}}^{2}\right]=E\left[\frac{A}{g\left(L\right)}{\left\{Y-m\left(Z\right)\right\}}^{2}\right],$$

where *g*(*L*) = *P*(*A* = 1|*L*). Alternatively, we can rewrite

$$\begin{array}{ll} E\left[{\left\{Y^{1}-m\left(Z\right)\right\}}^{2}\right] & =E\left[A{\left\{Y^{1}-m\left(Z\right)\right\}}^{2}+\left(1-A\right){\left\{Y^{1}-m\left(Z\right)\right\}}^{2}\right]\\ & =E\left(A{\left\{Y-m\left(Z\right)\right\}}^{2}+\left(1-A\right)E\left[{\left\{Y-m\left(Z\right)\right\}}^{2}|A=1,L\right]\right). \end{array}$$

This delivers a loss function that is not easy to optimize, in view of which we rewrite

$$E\left[{\left\{Y-m\left(Z\right)\right\}}^{2}|A=1,L\right]=\mathrm{Var}\left(Y|A=1,L\right)+{\left\{Q\left(L\right)-m\left(Z\right)\right\}}^{2},$$

where $Q\left(L\right)=E\left(Y|A=1,L\right)$ . Because the first term on the righthand side does not depend on *m*(.), minimization of (1) is then equivalent to minimization of

$$E\left(A{\left\{Y-m\left(Z\right)\right\}}^{2}+\left(1-A\right){\left\{Q\left(L\right)-m\left(Z\right)\right\}}^{2}\right).$$

The above identities suggest finding the function *m*(.) that minimizes

(2) $$\left[\frac{1}{n}\overset{n}{\underset{i=1}{\sum }}\frac{A_{i}}{g\left(L_{i}\right)}{\left\{Y_{i}-m\left(Z_{i}\right)\right\}}^{2}\right]+r\left(m\right),$$

or

(3) $$\left[\frac{1}{n}\overset{n}{\underset{i=1}{\sum }}{\left\{A_{i}Y_{i}+\left(1-A_{i}\right)Q\left(L_{i}\right)-m\left(Z_{i}\right)\right\}}^{2}\right]+r\left(m\right),$$

where *r*(.) is a penalty term that depends on the complexity of *m*(.). If *g*(.) and *Q*(.) were known, then this could be done by either weighting existing machine learning algorithms, or applying them to outcomes imputed as *A*_*i*_ *Y*_*i*_ + (1 − *A*_*i*_)*Q*(*L*_*i*_). When *g*(.) and *Q*(.) are unknown, we may substitute them by predictions $\hat{g}\left(.\right)$ and $\hat{Q}\left(.\right)$ , respectively, e.g., obtained via machine learning. We may then instead consider minimisation of

(4) $$\left[\frac{1}{n}\sum_{i=1}^{n}\frac{A_{i}}{\hat{g}\left(L_{i}\right)}{\left\{Y_{i}-m\left(Z_{i}\right)\right\}}^{2}\right]+r\left(m\right)$$

or

(5) $$[\frac{1}{n}\overset{n}{\underset{i=1}{\sum }}{\left\{A_{i}Y_{i}+\left(1-A_{i}\right)\hat{Q}\left(L_{i}\right)-m\left(Z_{i}\right)\right\}}^{2}]+r\left(m\right).$$

Here, the minimizer of (4) has been termed IPW-learner (see e.g. [19]); we will refer to the minimizer of (5) as a (non-orthogonal) imputation-learner.

Unfortunately, minimisation of (4) and (5) will not generally deliver an estimator of *m*(.) that is equivalent (in large samples) to the solution to (2) and (3), respectively. For instance, for (4), this is because

$$\begin{array}{ll} \frac{1}{n}\overset{n}{\underset{i=1}{\sum }}\frac{A_{i}}{\hat{g}\left(L_{i}\right)}{\left\{Y_{i}-m\left(Z_{i}\right)\right\}}^{2} & =\frac{1}{n}\overset{n}{\underset{i=1}{\sum }}\frac{A_{i}}{g\left(L_{i}\right)}{\left\{Y_{i}-m\left(Z_{i}\right)\right\}}^{2}+\frac{1}{n}\overset{n}{\underset{i=1}{\sum }}\{\frac{1}{\hat{g}\left(L_{i}\right)}-\frac{1}{g\left(L_{i}\right)}\}A_{i}{\left\{Y_{i}-m\left(Z_{i}\right)\right\}}^{2}, \end{array}$$

where the second term in the righthand side converges to zero, but may be sizeable in finite samples when the machine learning predictions $\hat{g}\left(L_{i}\right)$ are slowly converging. That this may be problematic can be seen, for instance, upon choosing $m\left(Z_{i}\right)=Z_{i}^{\prime }\beta$ . To understand the behavior of the resulting minimizer for *β*, we note that it is driven by the behavior of the derivative of the above identity w.r.t. *β*:

$$\begin{array}{ll} \frac{-2}{n}\overset{n}{\underset{i=1}{\sum }}\frac{A_{i}}{\hat{g}\left(L_{i}\right)}\left\{Y_{i}-m\left(Z_{i}\right)\right\}Z_{i} & =\frac{-2}{n}\overset{n}{\underset{i=1}{\sum }}\frac{A_{i}}{g\left(L_{i}\right)}\left\{Y_{i}-m\left(Z_{i}\right)\right\}Z_{i}-\frac{2}{n}\overset{n}{\underset{i=1}{\sum }}\{\frac{1}{\hat{g}\left(L_{i}\right)}-\frac{1}{g\left(L_{i}\right)}\}A_{i}\left\{Y_{i}-m\left(Z_{i}\right)\right\}Z_{i}. \end{array}$$

The first term on the right is *O*_*p*_(*n* ^−1/2^) at the true value *β*_0_, whereas the absolute value of each (say, the *j*th) component of the second term is upper bounded by

$$2{\left[\frac{1}{n}\overset{n}{\underset{i=1}{\sum }}{\left\{\frac{\hat{g}\left(L_{i}\right)-g\left(L_{i}\right)}{\hat{g}\left(L_{i}\right)g\left(L_{i}\right)}\right\}}^{2}\right]}^{1/2}{\left[\frac{1}{n}\overset{n}{\underset{i=1}{\sum }}A_{i}{\left\{Y_{i}-m\left(Z_{i}\right)\right\}}^{2}Z_{ij}^{2}\right]}^{1/2},$$

by the Cauchy-Schwarz inequality. Here, the second term is *O*_*p*_(1) and the first term will generally be *O*_*p*_(*n* ^−*b*^) for *b* < 1/2 when flexible, data-adaptive methods are used for *g*(.) (see e.g., [6]). This can make the second term in the above expansion dominant, causing the minimizer of (4) to be further than the typical *n* ^1/2^ distance away from *β*_0_, despite the use of a parametric model for *m*(.). Likewise, when more general data-adaptive meta-learners are used for *m*(.), their convergence rate may be harmfully affected by slow convergence in $\hat{g}\left(.\right)$ [12], 15], 16], which may well be much slower than the convergence rate of $\hat{m}\left(.\right)$ (at known nuisance parameters), especially when *L* is of higher dimension than *Z*.

### 2.2 Constructing orthogonal loss functions

The above concerns can be remedied by instead minimizing a double robust estimator of the (empirical) counterfactual prediction error [12], [20], [21], [22]:

$$\begin{array}{ll} & \frac{1}{n}\overset{n}{\underset{i=1}{\sum }}\frac{A_{i}}{\hat{g}\left(L_{i}\right)}\left({\left\{Y_{i}-m\left(Z_{i}\right)\right\}}^{2}-\hat{E}\left[{\left\{Y_{i}-m\left(Z_{i}\right)\right\}}^{2}|A_{i}=1,L_{i}\right]\right)\\ & +\hat{E}\left[{\left\{Y_{i}-m\left(Z_{i}\right)\right\}}^{2}|A_{i}=1,L_{i}\right]. \end{array}$$

By the earlier remarks, this is equivalent to minimization of

(6) $$\begin{array}{ll} & \frac{1}{n}\overset{n}{\underset{i=1}{\sum }}\frac{A_{i}}{\hat{g}\left(L_{i}\right)}[{\left\{Y_{i}-m\left(Z_{i}\right)\right\}}^{2}-{\left\{\hat{Q}\left(L_{i}\right)-m\left(Z_{i}\right)\right\}}^{2}]+{\left\{\hat{Q}\left(L_{i}\right)-m\left(Z_{i}\right)\right\}}^{2}=\frac{1}{n}\overset{n}{\underset{i=1}{\sum }}\frac{A_{i}}{\hat{g}\left(L_{i}\right)}{\left\{Y_{i}-m\left(Z_{i}\right)\right\}}^{2}+\{1-\frac{A_{i}}{\hat{g}\left(L_{i}\right)}\}{\left\{\hat{Q}\left(L_{i}\right)-m\left(Z_{i}\right)\right\}}^{2}=\frac{1}{n}\overset{n}{\underset{i=1}{\sum }}{\left\{A_{i}Y_{i}+\left(1-A_{i}\right)\hat{Q}\left(L_{i}\right)-m\left(Z_{i}\right)\right\}}^{2}+\frac{1}{n}\overset{n}{\underset{i=1}{\sum }}A_{i}\frac{1-\hat{g}\left(L_{i}\right)}{\hat{g}\left(L_{i}\right)}[{\left\{Y_{i}-m\left(Z_{i}\right)\right\}}^{2}-{\left\{\hat{Q}\left(L_{i}\right)-m\left(Z_{i}\right)\right\}}^{2}], \end{array}$$

where minimization of the latter is equivalent to minimization of

(7) $$\frac{1}{n}\overset{n}{\underset{i=1}{\sum }}{\left\{A_{i}Y_{i}+\left(1-A_{i}\right)\hat{Q}\left(L_{i}\right)-m\left(Z_{i}\right)\right\}}^{2}-A_{i}\frac{1-\hat{g}\left(L_{i}\right)}{\hat{g}\left(L_{i}\right)}\{Y_{i}-\hat{Q}\left(L_{i}\right)\}2m\left(Z_{i}\right).$$

Here, (6) shows that this double robust estimator updates the inverse probability weighted loss (4) to include also data for the unexposed (i.e., those with *A*_*i*_ = 0), thereby increasing efficiency and robustness to a possible lack of consistency of $\hat{g}\left(.\right)$ (provided that a consistent and sufficiently fast converging estimator $\hat{Q}\left(.\right)$ is used). Likewise, (7) shows that this double robust estimator updates the regression imputed loss (5) to increase robustness to a possible lack of consistency of $\hat{Q}\left(.\right)$ (provided that a consistent and sufficiently fast converging estimator $\hat{g}\left(.\right)$ is used). That minimization of (6) makes the meta-learner less sensitive to the estimation of nuisance parameters follows from ref. [12] (see also [19]); the crux of the idea is summarized in Appendix A. In particular, double robustness of the loss makes it Neyman-orthogonal - referred to as ‘orthogonal’ hereafter – in the sense that its directional derivatives w.r.t. *m*(.) and one of the nuisance parameters (e.g., $E\left(Y_{i}|A_{i}=1,L_{i}\right)$ or *P*(*A*_*i*_ = 1|*L*_*i*_)) have mean zero (at the truth).

### 2.3 Minimizing the orthogonal loss

Unfortunately, standard learners do not readily lend themselves towards minimization of (6) and (7). Ref. [15] remedies this by instead regressing the pseudo outcome

$$\frac{A_{i}}{\hat{g}\left(L_{i}\right)}\left\{Y_{i}-\hat{Q}\left(L_{i}\right)\right\}+\hat{Q}\left(L_{i}\right)$$

onto *Z*_*i*_ using a standard learner, i.e., by minimizing

$$\left(\frac{1}{n}\overset{n}{\underset{i=1}{\sum }}{\left[\frac{A_{i}}{\hat{g}\left(L_{i}\right)}\left\{Y_{i}-\hat{Q}\left(L_{i}\right)\right\}+\hat{Q}\left(L_{i}\right)-m\left(Z_{i}\right)\right]}^{2}\right)+r\left(m\right).$$

Related, but different proposals are given in [16], 17], 19]. A limitation of some proposals is that it is not always readily clear what counterfactual loss (e.g., (1)) they are aiming to minimize (see ref. [19] for further discussion on this point). Moreover, unlike (6), the above loss function contrasts the predictions *m*(*Z*_*i*_) with transformed outcomes, which may not belong to the same outcome space. This is especially problematic for dichotomous exposures, as it makes it difficult to guarantee estimated outcome probabilities in the unit interval. This is likewise the case for the above alternative proposals.

When *Z*_*i*_ = *L*_*i*_, this problem is readily accommodated by minimizing (4) instead. The reason is that, interestingly, this loss is orthogonal when *Z*_*i*_ = *L*_*i*_, though not otherwise. This can be seen because its directional derivative w.r.t. *m*(*Z*_*i*_) and then $\hat{g}\left(L_{i}\right)$ equals

$$\frac{1}{n}\overset{n}{\underset{i=1}{\sum }}A_{i}\left\{Y_{i}-m\left(Z_{i}\right)\right\}\theta \left(L_{i}\right)$$

for some function *θ*(*L*_*i*_); this has mean zero at the truth, since then *m*(*Z*_*i*_) = *E*(*Y*_*i*_|*A*_*i*_ = 1, *L*_*i*_). Informally, the reason that this works is that *m*(.) equals *Q*(.) in that case, so that the double robustness guarantee can also be achieved via *m*(.) rather than *Q*(.). Note that this is not the case when instead minimizing (5), which can be informally seen because it ignores the propensity score, or when minimizing

$$\left[\frac{1}{n}\overset{n}{\underset{i=1}{\sum }}A_{i}{\left\{Y_{i}-m\left(Z_{i}\right)\right\}}^{2}\right]+r\left(m\right),$$

which instead targets minimization of $E\left[{\left\{Y^{1}-m\left(Z\right)\right\}}^{2}|A=1\right]$ . In the next section, we will generalize this ad hoc solution (i.e., minimization of (4)) to make it work also when *Z*_*i*_ ⊆ *L*_*i*_. We will achieve this by targeting the estimation of the nuisance parameters.

## 3 i-learner

### 3.1 Proposal

We will find a solution to the above problem by minimizing (5) in a way that makes it equivalent to minimizing (7). For this, we wish the term

(8) $$\frac{1}{n}\overset{n}{\underset{i=1}{\sum }}A_{i}\frac{1-\hat{g}\left(L_{i}\right)}{\hat{g}\left(L_{i}\right)}\{Y_{i}-\hat{Q}\left(L_{i}\right)\}m\left(Z_{i}\right)$$

in (7) to be sufficiently close to zero for ‘all’ functions *m*(.) in the function class Γ (considering that *m*(.) is unknown). While it is generally close to zero (as a result of averaging contributions $Y_{i}-\hat{Q}\left(L_{i}\right)$ with mean zero (conditional on *L*_*i*_) in large samples), it is not generally close enough (as discussed in Section 2.1). In view of this, we will target or update the obtained predictions $\hat{Q}\left(L_{i}\right)$ to shrink (8) closer to zero, so that minimization of (5) based on the targeted predictions $\hat{Q}\left(.\right)$ is asymptotically equivalent to minimization of the orthogonal loss (7). This is inspired by targeted learning algorithms [5], but here necessitates targeting in infinitely many directions over the function class of *m*(.); targeting in infinitely many directions was previously considered in a longitudinal context in ref. [23]. We will refer to the resulting learner, which minimizes (5) based on the targeted predictions, as i-learner or imputation-learner.

Targeting $\hat{Q}\left(L_{i}\right)$ (i.e., updating initial estimates of $\hat{Q}\left(L_{i}\right)$ ) so that (8) is close to zero for all functions *m*(.) is challenging by the fact that *m*(.) is unknown. We will therefore make 2 assumptions. First, we will make a sparsity assumption that *m*(.) depends only on *D* ≤ *d* components of *Z*. Second, we will impose a smoothness assumption that *m*(.) obeys a Tensor product space model [24], which postulates that, on the scale of a known link function *h*(.), *m*(.) can be written as a finite sum:

(9) $$h\left\{m\left(Z\right)\right\}=\overset{J_{n}}{\underset{j=1}{\sum }}b_{j}\left(Z\right)\gamma_{j},$$

of *d*-dimensional products *b*_*j*_(.) (with *d* the dimension of *Z*) of univariate functions in a first-order Sobolev space (i.e., functions that are absolutely continuous and have a first order derivative that is mean square integrable); here, *h*(.) is the identity function (*h*(*x*) = *x*) for a continuous outcome, or the logistic function (*h*(*x*) = logit(*x*)) for a dichotomous outcome, and *J*_*n*_ may be chosen to be larger in larger sample sizes (see details later). For this, we will use products *b*_*j*_(.) of finite numbers of univariate cosine basis functions (including the constant function 1) to construct a richer function class (with unravelling rules as detailed in ref. [25] to (a) impose an ordering on the basis functions whereby lower order terms are prioritized and (b) exclude products of functions of more than *D*′ variables, where *D*′ is chosen by the user and assumed to exceed *D*). When *h*(.) is the identity link, the above smoothness assumption is approximately equivalent to all mixed first order derivatives of *m*(.) being mean square integrable [25]. In that case, which we will study in detail, it enables us to write *m*(.) as an infinite linear combination of (orthonormal) basis functions *b*(.), i.e.,

$$m\left(Z\right)=\overset{\infty }{\underset{j=1}{\sum }}b_{j}\left(Z\right)\gamma_{j},$$

with coefficients *γ*_*j*_ that decay at faster rate than *j* ^−1.5^ so that

$$\overset{\infty }{\underset{j=1}{\sum }}{\left(\frac{j}{\max\left(\log^{D-1}\;j,1\right)}\right)}^{2}\gamma_{j}^{2}\leq Q$$

for some constant *Q*. This exponential decay of the coefficient series *γ*_*j*_ justifies approximating *m*(*Z*) with the truncated series (9). Note that when these restrictions fail to hold, then by the choice of loss function, the i-learner will still provide the function with the smallest counterfactual prediction error in the considered (truncated) function class.

If (9) provides a good approximation, then the problem of shrinking (the absolute value of) (8) with *m*(.) unknown simplifies to that of shrinking (the absolute value of)

$$\frac{1}{n}\overset{n}{\underset{i=1}{\sum }}A_{i}\frac{1-\hat{g}\left(L_{i}\right)}{\hat{g}\left(L_{i}\right)}\{Y_{i}-\hat{Q}\left(L_{i}\right)\}b_{j}\left(Z_{i}\right)$$

for *j* = 1, …, *J*_*n*_ with *b*_*j*_(.) known. For this, we will build a parametric submodel around initial predictions ${\hat{Q}}^{\left(0\right)}\left(L_{i}\right)$ as follows:

$$h\left\{E\left(Y_{i}|A_{i}=1,L_{i}\right)\right\}=h\left\{{\hat{Q}}^{\left(0\right)}\left(L_{i}\right)\right\}+\epsilon^{\prime }b\left(Z_{i}\right)\frac{1-\hat{g}\left(L_{i}\right)}{\hat{g}\left(L_{i}\right)}$$

and fit the model using maximum likelihood with *l*_1_-penalisation with lasso penalty of the standard order $\sqrt{\log\left(J_{n}\right)/n}$ . The following theorem then shows that this procedure indeed achieves the required shrinkage.

**Proposition 1.**

Let *h*(.) be the identity function and suppose that the just discussed sparsity and smoothness assumptions hold. Let the penalty term *λ* be of the order $O_{p}\left(\sqrt{\log\;J_{n}/n}\right)$ with *J*_*n*_ = *C*(*D*)*d* ^*D*′^ *n* ^1/3^ log^*D*′−1^(*n*) and *C*(*D*) a constant that may depend on *D*. Assume that the density *f*_*Z*_(.) of *Z* and the conditional variance Var(*ϵ*|*Z*) are bounded, with $\epsilon \equiv A_{i}\left\{1/\hat{g}\left(L_{i}\right)-1\right\}\left\{Y_{i}-Q\left(L_{i}\right)\right\}$ (with $\hat{g}\left(.\right)$ considered fixed). Then

$$\frac{1}{n}\overset{n}{\underset{i=1}{\sum }}\overset{\infty }{\underset{j=J_{n}+1}{\sum }}A_{i}\frac{1-\hat{g}\left(L_{i}\right)}{\hat{g}\left(L_{i}\right)}\{Y_{i}-\hat{Q}\left(L_{i}\right)\}b_{j}\left(Z_{i}\right)\gamma_{j}$$

is of the order

(10) $$O_{p}\left(\log^{\frac{D-1}{3}}\;n\left\{\log J_{n}n^{-2/3}\;\log^{\frac{D-1}{3}}\;n+n^{-5/6}+n^{-b-1/3}\right\}\right).$$

□

This Proposition confirms that the term (8) shrinks from being of the order *O*_*p*_(*n* ^−*b*^) to being (10). Here, the last 2 terms reflect approximation error as a result of approximating *m*(.) by means of a finite number, *J*_*n*_, of basis functions. It shows that the earlier root mean squared prediction error in ${\hat{Q}}^{\left(0\right)}$ of the order *O*_*p*_(*n* ^−*b*^) shrinks by a factor *n* ^−1/3^, up to polylog terms (note also that the effective dimension *D* only shows up on the exponent of the log sample size, rather than the sample size itself). The first term arises from not knowing which *D* out of *d* terms to select and is of the order *O*_*p*_(*n* ^−2/3^), up to polylog terms. This term is not dependent on the convergence rate of ${\hat{Q}}^{\left(0\right)}$ , as the procedure is effectively trying to set (8) to zero no matter the choice of ${\hat{Q}}^{\left(0\right)}$ , and depends only on the logarithm of the ambient dimension *d*. Proposition 1 enables us to bound the (part of the) mean squared error of the proposed estimator $\hat{m}\left(Z\right)$ in Theorem 1.

**Theorem 1.**

Assume that $|Y-\hat{Q}\left(L\right)|\leq M$ for *M* > 0 with probability 1. The mean squared error of the proposed estimator $\hat{m}\left(Z\right)$ can then be upper bounded, for arbitrary *δ*_1_, *δ*_2_ > 0, as

$$\begin{array}{ll} E[{\left\{\hat{m}\left(Z\right)-m\left(Z\right)\right\}}^{2}] & \leq {\left(1-\delta_{1}-\delta_{2}\right)}^{-1}\left(\underset{\eta }{\sup}|L\left(m,\eta \right)-L\left(\hat{m},\eta \right)\right|\;+\frac{1}{\delta_{1}}E\left(\frac{g^{2}\left(L\right)}{{\bar{g}}^{6}\left(L\right)}{\left\{Q\left(L\right)-\bar{Q}\left(L\right)\right\}}^{2}{\left\{\hat{g}\left(L\right)-g\left(L\right)\right\}}^{4}\right)\;+\frac{1}{\delta_{2}}E(\frac{g^{2}\left(L\right)}{{\bar{g}}^{4}\left(L\right)}{\left\{\hat{g}\left(L\right)-g\left(L\right)\right\}}^{2}{\left\{\hat{Q}\left(L\right)-Q\left(L\right)\right\}}^{2})\;\;-\frac{2}{n}\overset{n}{\underset{i=1}{\sum }}[\frac{A_{i}\{1-\hat{g}\left(L_{i}\right)\}}{\hat{g}\left(L_{i}\right)}\{Y_{i}-\hat{Q}\left(L_{i}\right)\}\{\hat{m}\left(Z_{i}\right)-m\left(Z_{i}\right)\}]), \end{array}$$

where

$$L\left(m,\eta \right)\equiv E\left[\frac{A}{g\left(L\right)}{\left\{Y-m\left(Z\right)\right\}}^{2}+\left\{1-\frac{A}{g\left(L\right)}\right\}{\left\{Q\left(L\right)-m\left(Z\right)\right\}}^{2}\right]$$

for *η* ≡ (*g*, *Q*), $\bar{g}\left(L\right)=tg\left(L\right)+\left(1-t\right)\tilde{g}\left(L\right)$ for some *t* ∈ [0, 1] and $\tilde{g}\left(L\right)$ an element of a vector space large enough to contain $\hat{g}\left(L\right)$ and, likewise, $\bar{Q}\left(L\right)=tQ\left(L\right)+\left(1-t\right)\tilde{Q}\left(L\right)$ for $\tilde{Q}\left(L\right)$ an element of a vector space large enough to contain $\hat{Q}\left(L\right)$ .

Here, the first component is the oracle excess risk (if nuisance parameters were known), which we expect to be of the order *O*_*p*_(*n* ^−2/3^), up to polylog terms (see lemma C.6 in [25]). Theorem 1 (whose proof is in Appendix B) thus shows that the mean squared error is upper bounded by the excess risk plus (10), which is generally of the same order of magnitude, plus a product rate term involving the mean squared errors of the nuisance parameter estimators $\hat{g}\left(.\right)$ and $\hat{Q}\left(.\right)$ . This confirms that optimization of (7) roughly delivers oracle behavior, and thus that the considered targeting step suffices. In particular, the slow convergence rates in the nuisance parameter estimators are attenuated (by the product rate term, which results from the loss function being approximately Neyman-orthogonal, and by the targeting step) before propagating into the meta-learner. One exception is when parametric regression is used for *m*(.); however, in that case, one can make (8) exactly zero by letting *b*(*Z*) be the derivatives of *m*(*Z*) w.r.t. the parameters indexing its parametric model (without the need for *l*_1_-penalization).

### 3.2 Cross-fitting

As in refs. [12], 15], 16], sample-splitting is crucial to theoretically justify adequate performance of the i-learner. Through sample-splitting we obtain the nuisance parameter estimates from a sample that is independent from the sample used for plug-in empirical risk minimization, and therefore we can treat the nuisance parameters in the empirical risk minimization problem as fixed. This allows us to invoke the performance guarantees for the machine learning algorithms used for the empirical risk minimization, since it requires the data to be independent and identically distributed (i.i.d.). To prevent efficiency loss, we performed sample splitting via a cross-fitting procedure as follows. First, we split the data on the *K* (e.g. 5) disjoint, roughly equally sized folds. Then for each fold *k* = 1, …, *K*, we train *E*(*A*|*L*) and *E*(*Y*|*A* = 1, *L*) on the data from the *K* − 1 remaining folds, and subsequently target the predictions for *E*(*Y*|*A* = 1, *L*) on those same folds (we chose not to do the targeting step on the *k*th fold in view of the high-dimensionality of the models used for targeting). We then used the resulting predictions for *E*(*Y*|*A* = 1, *L*) to calculate imputed outcomes in the *k*th fold. After having repeated this for all folds, we regressed the resulting imputed outcomes on the covariates *Z* across all folds in one go.

## 4 Simulation study

We have evaluated the performance of the proposal in two simulation experiments. Our first simulation study focuses on continuous outcomes using the following data generating mechanism (largely) from ref. [26] to generate i.i.d. data: $L_{i}∼N\left(0,\Sigma_{d\times d}\right)$ , where Σ_*d*×*d*_ is a *d*-dimensional correlation matrix with *d* = 20 and correlations drawn from a normal distribution and varying from −0.5 to 0.5,

$$\begin{array}{ll} A_{i}|L_{i} & ∼\text{Bern}\left\{\pi \left(L_{i}\right)\right\}\\ \pi \left(L_{i}\right) & =1/\left[1+\exp\left\{V_{1}-0.5V_{2}+0.25V_{3}+0.1V_{4}\right\}\right]\\ b\left(L_{i}\right) & =210+27.4V_{1}+13.7V_{2}+13.7V_{3}+13.7V_{4}, \end{array}$$

with

$$\begin{array}{ll} V_{1} & =\exp\left(L_{1}/2\right)\\ V_{2} & =L_{2}/\left\{1+\exp\left(L_{1}\right)\right\}+10\\ V_{3} & ={\left(L_{1}L_{3}/25+0.6\right)}^{3}\\ V_{4} & ={\left(L_{2}+L_{4}+20\right)}^{2}, \end{array}$$

and $Y_{i}=Y_{i}^{a}|L_{i}∼N\left\{b\left(L_{i}\right),1\right\}$ for *a* = 0, 1. Our second simulation study focuses on dichotomous outcomes, using the following data generating mechanism (largely) from ref. [27]. Covariates *L*_*i*_ were generated as before,

$$\begin{array}{ll} A_{i}|L_{i} & ∼\text{Bern}\left\{\pi \left(L_{i}\right)\right\}\\ \pi \left(L_{i}\right) & =1/\left[1+\exp\left\{2+2\;\sin\left(K_{i}\right)+2\;\cos\left(K_{i}\right)\right\}\right]\\ b\left(L_{i}\right) & =1/\left[1+\exp\left\{-2.5+2\;\cos^{2}\left(K_{i}^{*}\right)\right\}\right] \end{array}$$

with $K_{i}=\sum_{j=1}^{p}L_{ij}/j$ , $K_{i}^{*}=\sum_{j=1}^{p}L_{ij}/\left(p-j+1\right)$ and $Y_{i}=Y_{i}^{a}|L_{i}∼\text{Bern}\left\{b\left(L_{i}\right)\right\}$ for *a* = 0, 1.

In each of 500 replications, for different estimators $\hat{m}\left(.\right)$ of *m*(.), the simulation studies evaluate the mean squared errors

$$\frac{1}{500}\overset{500}{\underset{i=1}{\sum }}{\left\{\hat{m}\left(Z_{i}^{\left(v\right)}\right)-m\left(Z_{i}^{\left(v\right)}\right)\right\}}^{2}$$

based on a random validation sample of observations $Z_{i}^{\left(v\right)}$ , *i* = 1, …, 500, drawn from the same data-generation model as specified above. We then averaged mean squared errors over the 500 simulation experiments. Specifically, we considered the following learners of *E*(*Y* ^1^|*Z*), all based on *l*_1_-penalized sieves with cosine basis, 5-fold cross-fitting, identity link in simulation experiment 1 and logistic link in simulation experiment 2, and ‘standard’ learners (to be specified later) for the nuisance parameters: (1) naïve: sieves fitted in the treated subgroup; (2) IPW: sieves fitted in the treated subgroup, using inverse probability weighting as in (4); (3) imputation: sieves based on regression mean imputation, as in (5); (4) DR: DR-learner; (5) i-learner: sieves based on targeted regression mean imputations. In simulation experiment 2, DR-learner predictions were constrained to the unit interval (and labelled cDR). As standard learners for the estimation of nuisance parameters, we considered regression forests as well as SuperLearner with a library given by generalized linear main effect and interaction models, stepwise regression, regression forests and generalized additive models. We repeated this for *Z*_*i*_ equalling the first 2 covariates in *L*_*i*_, the first 5, and finally all 20. Note that DR-learner is based on unconstrained pseudo-outcomes and that estimator 1 is expected to be biased as a result of ignoring confounding. Estimators 2 and 3 are based on non-orthogonal learners (except estimator 2 when *V*_*i*_ = *L*_*i*_) and may therefore also be subject to bias. All other learners are orthogonal.

The results from the first simulation experiment (see Table 1) show favourable performance for the proposed imputation-learner and i-learner. Their performance is comparable with SuperLearner, and slightly worse for the i-learner when nuisance parameters are estimated via random forests. This may be due to the fact that ‘reasonable’ convergence of the random forest regression fits – as required for the i-learner to be orthogonal – is unlikely met in this complex data-generating mechanism, as opposed to when the SuperLearner is used for nuisance parameter estimation. Results for the (orthogonal) DR-learner are drastically worse, with the exception of random forests regression at large sample size and higher-dimensional *Z*. Unsurprisingly, mean squared errors drop drastically with the increasing number of predictors *Z*. The second simulation experiment (see Table 2) uses nuisance parameters that were previously observed to be well estimable using random forest regression [27]. Here, the best performance is found for the proposed imputation-learner, despite its lack of orthogonality. Results for the constrained (orthogonal) DR-learner are competitive with the i-learner when nuisance parameters are estimated via random forests, but drastically worse when the SuperLearner is used, due to the greater variability in the resulting nuisance parameter estimates. This is so, despite constraining over 30 % of counterfactual predictions that fell outside of the unit interval.

**Table 1:** Results of simulation experiment 1 (continuous outcome) at samples sizes 250 and 500. Choice of learner for the nuisance parameters (RF: random forests, SL: SuperLearner), the covariate dimension for the meta-learner, the number of basis functions used in the penalized sieve estimator, mean squared errors of the 5 listed meta-learners.

*n*	Learner	dim(*Z*)	*#* Basis	Naïve	IPW	Imputation	DR	i-learner
250	RF	2	10	910	898	568	710	603
		5	20	716	706	379	562	429
		20	50	688	683	379	626	432
	SL	2	10	851	855	439	464	435
		5	20	537	552	46	107	45
		20	50	474	514	21	106	21
500	RF	2	10	495	495	480	471	466
		5	20	105	100	128	102	120
		20	50	46	47	117	87	110
	SL	2	10	495	497	446	448	446
		5	20	105	102	39	43	39
		20	50	47	49	13	21	13

**Table 2:** Results of simulation experiment 2 (dichotomous outcome) at samples sizes 500 and 1,000. Choice of learner for the nuisance parameters (RF: random forests, SL: SuperLearner), the covariate dimension for the meta-learner, the number of basis functions used in the penalized sieve estimator, mean squared errors of the 5 listed meta-learners, and the percentage of DR-learner estimates outside of the unit interval (%). cDR refers to the DR-learner estimates, truncated to the unit interval.

*n*	Learner	dim(*Z*)	*#* Basis	Naïve	IPW	Imputation	cDR	i-learner	%
500	RF	2	10	0.025	0.026	0.017	0.021	0.024	1.8
		5	20	0.024	0.026	0.017	0.022	0.022	1.6
		20	50	0.025	0.026	0.016	0.021	0.022	1.6
	SL	2	10	0.025	0.051	0.017	0.068	0.028	31.7
		5	20	0.024	0.049	0.018	0.068	0.026	32.9
		20	50	0.025	0.049	0.017	0.065	0.025	32.7
1,000	RF	2	10	0.022	0.023	0.016	0.020	0.021	1.5
		5	20	0.021	0.022	0.016	0.020	0.020	1.0
		20	50	0.021	0.022	0.014	0.019	0.018	1.2
	SL	2	10	0.021	0.040	0.017	0.068	0.025	30.3
		5	20	0.021	0.041	0.017	0.068	0.023	30.4
		20	50	0.021	0.041	0.015	0.065	0.021	31.5

## 5 Causal prediction in critical care

Acute kidney injury (AKI) is an abrupt decrease in kidney function, which is commonly defined in terms of KDIGO criteria [28]. Renal replacement therapy (RRT) is a treatment that is commonly used for the management of critically ill patients with severe AKI, in particular those experiencing metabolic or fluid-related complications. RRT may rapidly correct some of the life-threatening complications associated with AKI, e.g., severe hyperkalaemia (i.e., serum potassium above 6.0 mmol/L), metabolic acidosis (i.e., pH below 7.2) or pulmonary oedema (i.e., abnormal accumulation of fluid in lungs due to fluid overload). However, it is a very invasive treatment and may put treated patients at risk of bleeding, infection, hemodynamic instability, electrolyte abnormalities, …It is therefore of paramount importance to carefully and appropriately judge the costs and benefits of initiating such an invasive intervention. As part of the development of a decision support system, we are therefore interested in predicting the 7-day ICU mortality under initiation of RRT within 24 h from the time of stage 2 AKI diagnosis in the stage ≥2 AKI patient population (i.e., the potential outcome *Y* ^1^), and the corresponding 7-day ICU mortality under no initiation of RRT (i.e., *Y* ^0^). For this, we analyzed data from the Intensive Care Information System of the Ghent University Hospital ICUs, which contains records from all adult patients admitted to the intensive care unit since 2013. In our analysis we considered 3,728 adult stage 2 and 3 AKI patients admitted to the ICU between 1/1/2013 and 31/12/2017, who had no recorded RRT history and no RRT restrictions by the time of the inclusion at stage 2 AKI diagnosis.

For each patient the database holds information on several characteristics, e.g., ICU admission time, ICU discharge time, vital status at discharge, timestamps of all dialysis sessions during each ICU episode, baseline covariates (e.g., age, weight, gender, admission category {“No surgery”, “Planned surgery”, “Emergency surgery”}, receipt of dialysis prior to current ICU admission, chronic kidney disease diagnosis prior to current ICU admission) and longitudinal measurements over the ICU episode (e.g., SOFA scores, having reached KDIGO AKI (stage 1/2/3) creatinine condition, having reached KDIGO AKI (stage 1/2/3) oliguric condition, receipt of diuretics, cumulative total fluid intake, cumulative total fluid output, arterial pH, serum potassium (in mmol/L), serum ureum (in mg/dL), serum magnesium (in mmol/L), fraction of inspired oxygen (FiO_2_), peripheral oxygen saturation (SpO_2_), arterial oxygen concentration (PaO_2_), ratio of arterial oxygen concentration to the fraction of inspired oxygen (P/F ratio), DNR (“Do Not Resuscitate”) code) and their timestamps.

We applied several meta-learners based on *l*_1_-penalized sieve regression with logistic link and cosine basis in order to predict potential outcomes based on the following subset of covariates *Z*: age on admission, gender, serum potassium, arterial pH, cumulative total fluid intake and cumulative total fluid output. In particular, we implemented the ‘naïve’ approach, the IPW-learner, the DR-learner, the proposed i-learner and (non-orthogonal) imputation learner. The models for the nuisance parameters have been computed using SuperLearner [29] with the following list of wrappers: glm, glmnet, random forest (ranger) and xgboost. To perform our analysis, we split the data into three equally sized parts: training set A, training set B and a test set. To make the most efficient use of the available data we apply cross-fitting as described in Section 3.2. In the final step, given the models for the mean potential outcomes obtained via cross-fitting on the training data sets A and B, we use the test data set to obtain the final output, i.e., 7-day ICU mortality under initiation of RRT (and similarly under no initiation of RRT) within 24 h from stage 2 AKI diagnosis, conditional on the selected patient characteristics. We evaluate performance on the test set to avoid possible overoptimism, which could arise once evaluating the performance of different methods on the same data that has been used for training the models.

**Figure 1:**
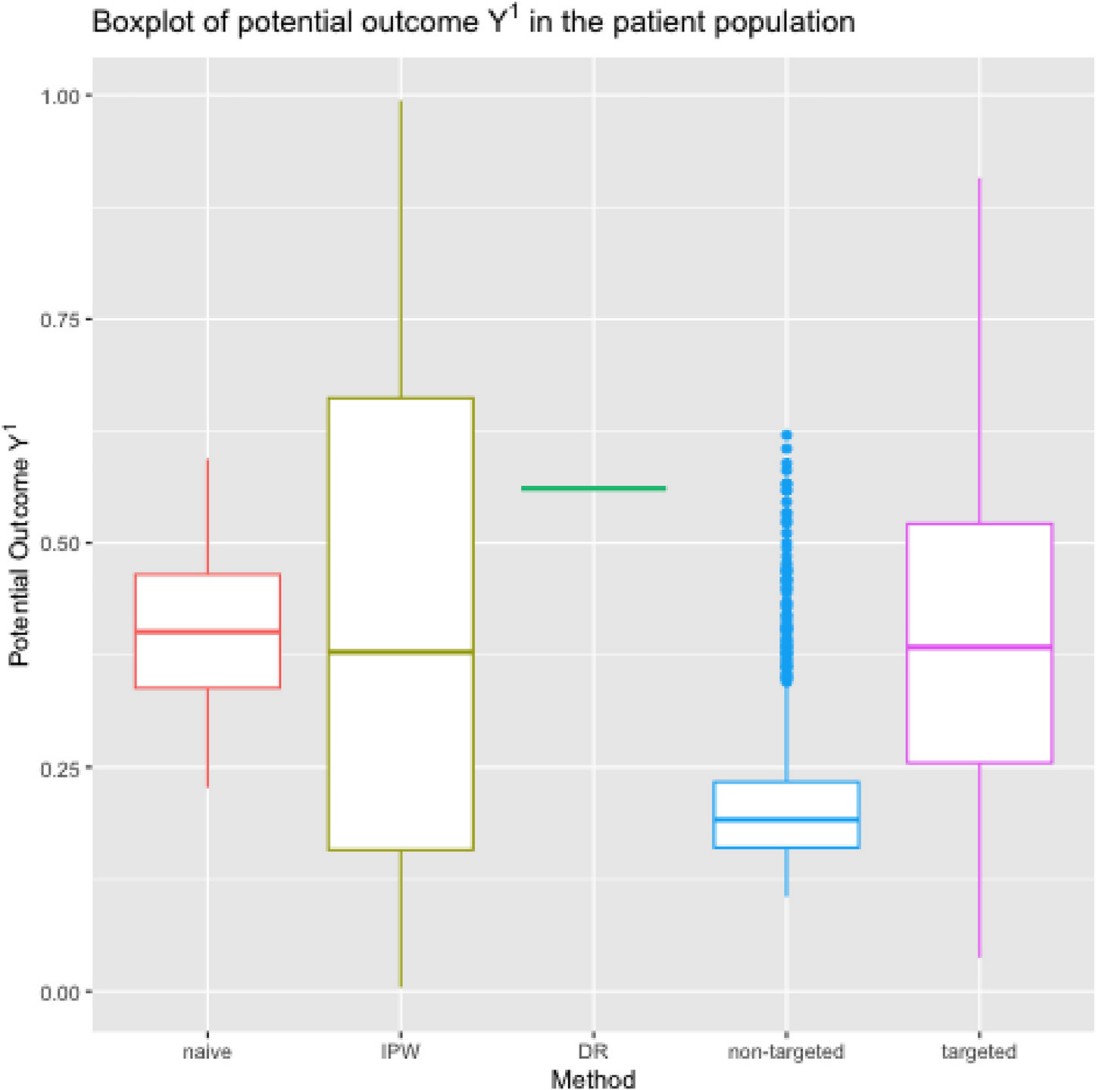
Boxplot of the 7-day ICU mortality under initiation of RRT within 24 h from the stage 2 AKI diagnosis in the stage ≥2 AKI patient population, i.e., the potential outcome *Y* ^1^, conditional on the values of age on admission, gender, serum potassium, arterial pH, fluid intake and fluid output computed in the whole patient population using “naive” approach (“naive”), IPW-learner (“IPW”), DR-learner (“DR”), proposed targeted learner, i.e. i-learner (“targeted”), and its non-targeted equivalent (“non-targeted”).

**Figure 2:**
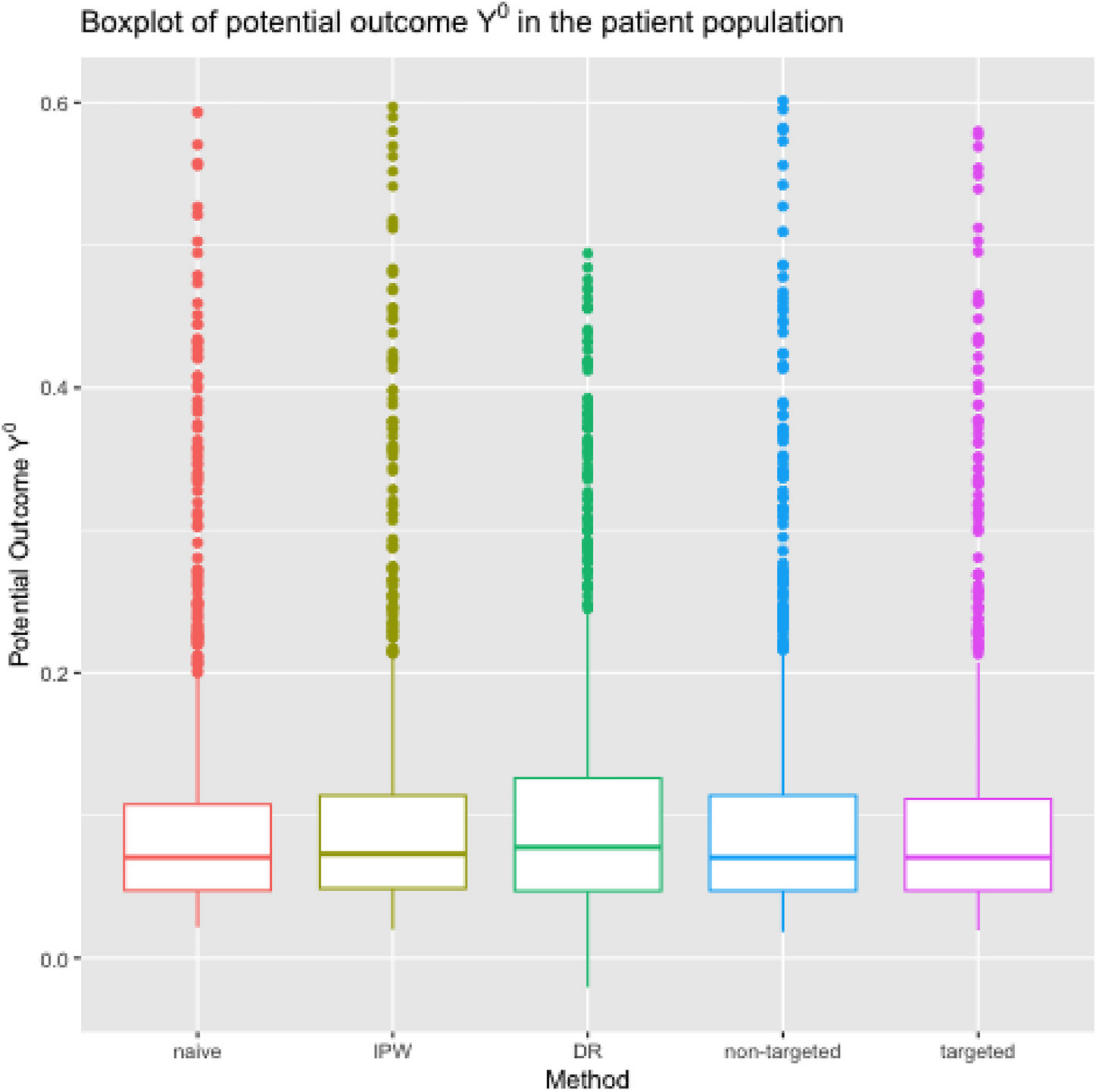
Boxplot of the 7-day ICU mortality without initiation of RRT within 24 h from the stage 2 AKI diagnosis in the stage ≥2 AKI patient population, i.e., the potential outcome *Y* ^0^, conditional on the values of age on admission, gender, serum potassium, arterial pH, fluid intake and fluid output computed in the whole patient population using “naive” approach (“naive”), IPW-learner (“IPW”), DR-learner (“DR”), proposed targeted learner, i.e. i-learner (“targeted”), and the proposed (non-orthogonal) imputation learner (“non-targeted”).

**Figure 3:**
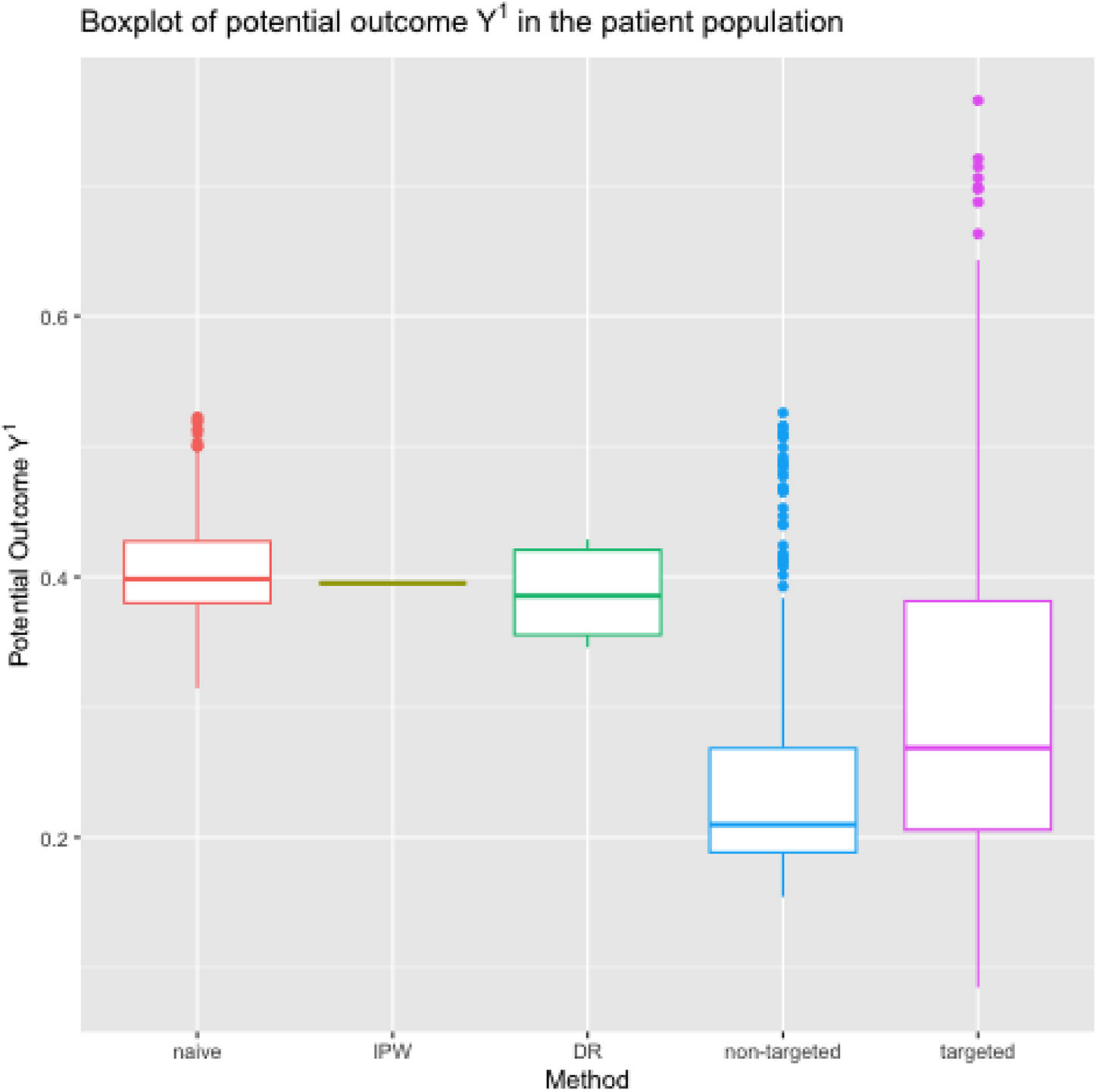
Boxplot of the 7-day ICU mortality under initiation of RRT within 24 h from the stage 2 AKI diagnosis in the stage ≥2 AKI patient population whose estimated propensity score exceeds 1 %, i.e., the potential outcome *Y* ^1^, conditional on the values of age on admission, gender, serum potassium, arterial pH, fluid intake and fluid output computed in the whole patient population using “naive” approach (“naive”), IPW-learner (“IPW”), DR-learner (“DR”), proposed targeted learner, i.e. i-learner (“targeted”), and its non-targeted equivalent (“non-targeted”).

Figure 1 presents a boxplot of the 7-day ICU mortality estimates under initiation of RRT within 24 h from stage 2 AKI diagnosis in stage ≥2 AKI patients. It shows the poor performance of DR-learner, which is the result of extreme propensity scores for some patients, making the pseudo-outcomes highly variable, thereby obscuring their association with covariates. In Appendix C, we show results for sieves with numbers of basis functions different from the default in the Sieve package [25]. It shows the lack of stability of some learners (in particular, IPW-learner and DR-learner) as opposed to the proposed i-learner and (non-orthogonal) imputation learner. Figure 2 shows analogous results for the 7-day ICU mortality without initiation of RRT within 24 h from the stage 2 AKI diagnosis in the stage ≥2 AKI patient population. Results are more comparable between learners because the majority of patients was not treated within 24 h from AKI-diagnosis. Because predicting *Y* ^1^ may be difficult for some patients, and even irrelevant for patients that clinicians would never consider for RRT, we repeated the analysis within the subset of patients whose estimated propensity score exceeds 1 %. The results in Figure 3 continue to show better performance for the proposed learners, which are also less sensitive to the chosen number of basis functions (see Appendix C).

## 6 Discussion

We have shown how non-orthogonal learners can be made approximately Neyman-orthogonal by invoking infinite-dimensional targeting procedures, akin to iTMLE [23]. This is justified according to our asymptotic approximations in terms of regret bounds, as well as confirmed to work well empirically. This result is useful because orthogonal learning procedures often demand optimizing loss functions that are difficult to optimize. Popular learners, such as DR-learner and R-learner, overcome this by applying standard learners to suitably transformed outcomes, possibly additionally invoking well-chosen observation weights. However, this comes at the expense of sub-optimal performance. We have instead remedied this by applying standard learners to carefully imputed (counterfactual) outcomes. Concurrent work by ref. [30] shows related results for conditional average treatment effects and more general functionals, but does not consider penalization.

We proposed two imputation estimators, one of which – referred to as the i-learner - has stronger theoretical justification due to its partial insulation against the possible poor quality of imputed outcomes. However, empirical simulation studies revealed that the simpler non-orthogonal imputation estimator often performed competitively or even better across a wide range of simulation settings, only some of which were detailed here. This observation might be related to the convergence rate of oracle learners (based on known nuisance parameters) already being slow, and thereby not being significantly impacted by the convergence rates of nuisance parameter estimators, a topic that warrants further investigation. Moreover, it is important to realize that our focus on Neyman-orthogonality can only partially explain the differences in performance between the different learners. These are additionally driven by differences in the performance of the oracle learners, as well as differences in the distribution of targeted versus non-targeted nuisance parameter estimators, a study of which is beyond the scope of this work. Finally, further work is needed to refine the selection of tuning parameters, which currently relies on standard software that does not account for the differences between imputed and real outcomes. This consideration is part of the reason we chose to impute counterfactual outcomes on treatment (control) only for subjects on control (treatment). Nevertheless, an analogous development could be made based on imputing outcomes for all, which would better align with standard targeted learning strategies, but is not *a priori* expected to deliver favourable behavior.

Our proposal provides a natural alternative for DR-learner, which likewise aims to minimize counterfactual prediction error [19], 22]. It remains to be seen how it extends to an R-learner-like and a ‘treatment effect in the untreated’ strategy for counterfactual prediction, which according to the findings in ref. [19], would naturally focus on minimizing

$$E\left[w\left(L\right){\left\{Y^{1}-m\left(Z\right)\right\}}^{2}\right],$$

with *w*(*L*) = *P*(*A* = 1|*L*)*P*(*A* = 0|*L*) or *w*(*L*) = *P*(*A* = 0|*L*), respectively. This could likewise be done by minimizing

$$\frac{1}{n}\overset{n}{\underset{i=1}{\sum }}\hat{g}\left(L_{i}\right)\{1-\hat{g}\left(L_{i}\right)\}{\left\{A_{i}Y_{i}+\left(1-A_{i}\right)\hat{Q}\left(L_{i}\right)-m\left(Z_{i}\right)\right\}}^{2}+r\left(m\right),$$

but necessitates a different targeting step, which we will develop in future work. Such further extension will be essential for our motivating application: there are many patients for whom RRT is not a meaningful treatment strategy, which makes it suboptimal to minimize counterfactual prediction error over the full stage ≥2 patient population.

Limitations of our work are that it imposes sparsity assumptions on the counterfactual means *E*(*Y* ^1^|*Z*) and *E*(*Y* ^0^|*Z*), and that we have restricted a formal study of error bounds to sieves in linear models. Sparsity assumptions may more likely hold w.r.t. the conditional causal effect *E*(*Y* ^1^ − *Y* ^0^|*Z*) because causal effects may often be homogeneous, small or even absent; in such cases, differences between our current predictions for *Y* ^1^ and *Y* ^0^ may show erratic behavior by not leveraging smoothness/sparsity assumptions directly on *E*(*Y* ^1^ − *Y* ^0^|*Z*) [31]. In future work, we will therefore study how this can be done when the aim is to predict both counterfactuals. We will then also develop insight into the number and choice of basis functions [18], and study how well the proposed targeting procedure continues to work when the targeting step is based on penalized sieves, but the final optimization of the loss function is based on more general learning procedures (e.g., random forest regression). Finally, drawing inference based on the obtained infinite-dimensional estimates *E*(*Y* ^1^|*Z*) and *E*(*Y* ^0^|*Z*) is a challenging problem that has received little attention so far, and for which we will study the use of debiased lasso [32], as well as more generic procedures [13], 33].

## Figures and Tables

**Figure 4:**
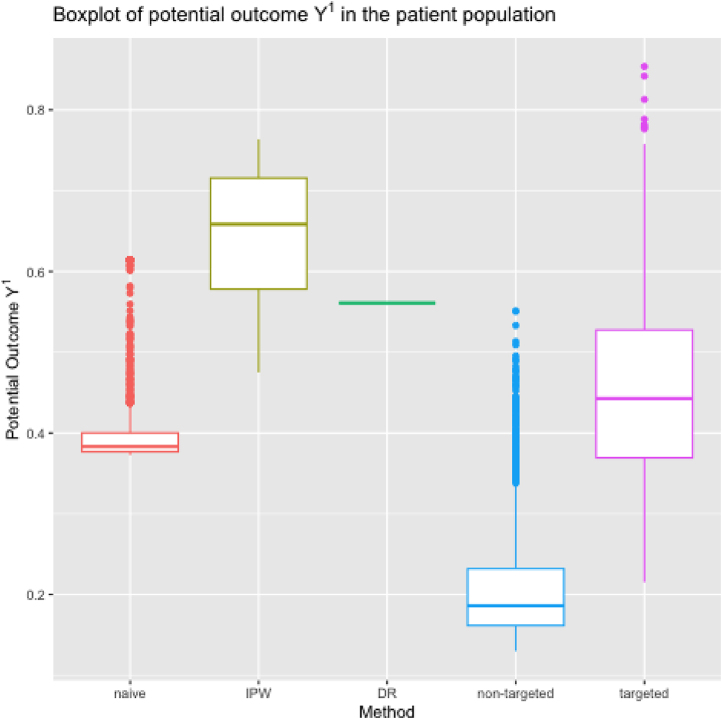
Boxplot of the 7-day ICU mortality under initiation of RRT within 24 h from the stage 2 AKI diagnosis in the stage ≥2 AKI patient population, i.e., the potential outcome *Y* ^1^, conditional on the values of age on admission, gender, serum potassium, arterial pH, fluid intake and fluid output computed in the whole patient population using “naive” approach (“naive”), IPW-learner (“IPW”), DR-learner (“DR”), proposed targeted learner, i.e. i-learner (“targeted”), and its non-targeted equivalent (“non-targeted”), using 20 basis functions.

**Figure 5:**
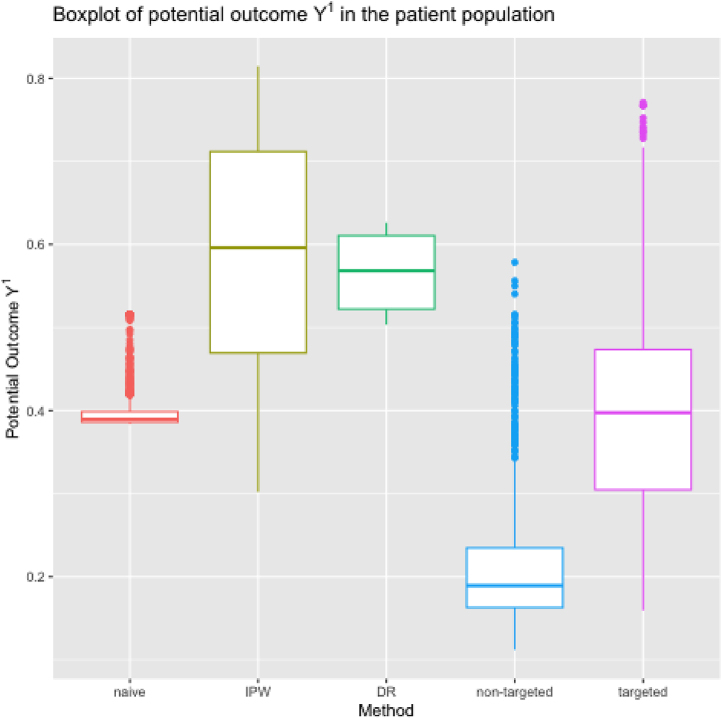
Boxplot of the 7-day ICU mortality under initiation of RRT within 24 h from the stage 2 AKI diagnosis in the stage ≥2 AKI patient population, i.e., the potential outcome *Y* ^1^, conditional on the values of age on admission, gender, serum potassium, arterial pH, fluid intake and fluid output computed in the whole patient population using “naive” approach (“naive”), IPW-learner (“IPW”), DR-learner (“DR”), proposed targeted learner, i.e. i-learner (“targeted”), and its non-targeted equivalent (“non-targeted”), using 50 basis functions.

**Figure 6:**
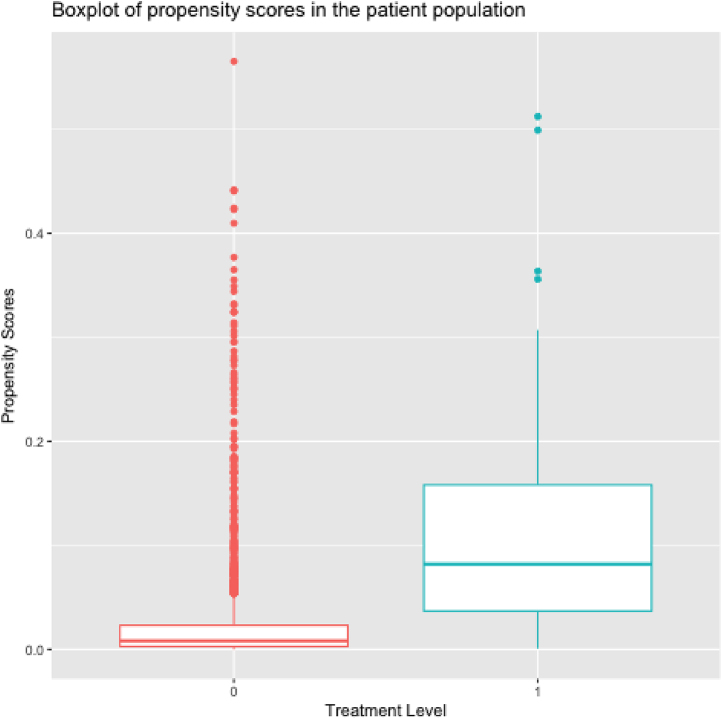
Boxplot of the propensity scores in both treatment groups. Treatment level “1” denotes “RRT initiation” and treatment level “0” denotes “no RRT initiation”.

**Figure 7:**
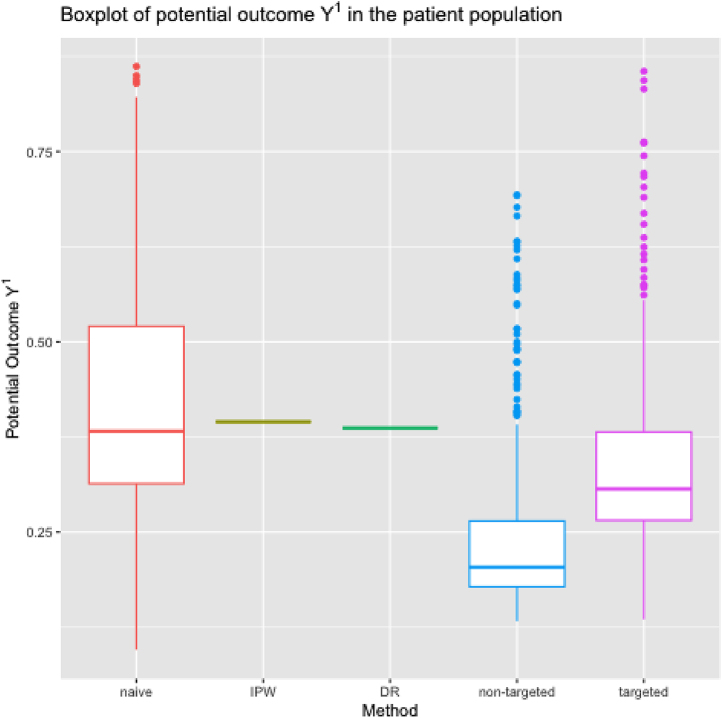
Boxplot of the 7-day ICU mortality under initiation of RRT within 24 h from the stage 2 AKI diagnosis in the stage ≥2 AKI patient population whose estimated propensity score exceeds 1 %, i.e., the potential outcome *Y* ^1^, conditional on the values of age on admission, gender, serum potassium, arterial pH, fluid intake and fluid output computed in the whole patient population using “naive” approach (“naive”), IPW-learner (“IPW”), DR-learner (“DR”), proposed targeted learner, i.e. i-learner (“targeted”), and its non-targeted equivalent (“non-targeted”), using 20 basis functions.

**Figure 8:**
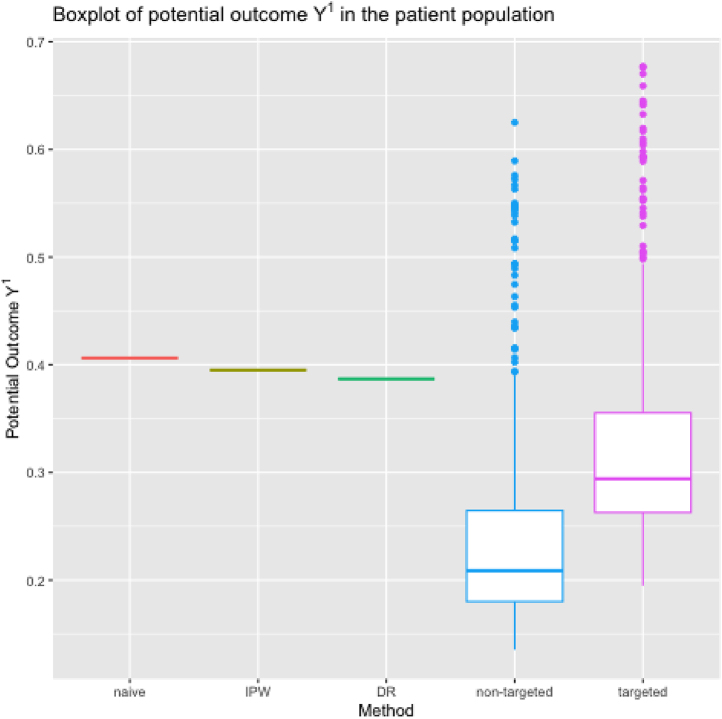
Boxplot of the 7-day ICU mortality under initiation of RRT within 24 h from the stage 2 AKI diagnosis in the stage ≥2 AKI patient population whose estimated propensity score exceeds 1 %, i.e., the potential outcome *Y* ^1^, conditional on the values of age on admission, gender, serum potassium, arterial pH, fluid intake and fluid output computed in the whole patient population using “naive” approach (“naive”), IPW-learner (“IPW”), DR-learner (“DR”), proposed targeted learner, i.e. i-learner (“targeted”), and its non-targeted equivalent (“non-targeted”), using 50 basis functions.

## Appendix A

In this Appendix, we develop oracle bounds for the (orthogonal) meta-learner which minimizes the double-robust loss

$$L\left(m,\eta \right)\equiv E\left[\frac{A}{g\left(L\right)}{\left\{Y-m\left(Z\right)\right\}}^{2}+\left\{1-\frac{A}{g\left(L\right)}\right\}{\left\{Q\left(L\right)-m\left(Z\right)\right\}}^{2}\right]$$

for *η* ≡ (*g*, *Q*).

A Taylor series expansion using directional derivatives [12] shows that

$$\begin{array}{ll} L\left(\hat{m},\hat{\eta }\right) & =L\left(m,\hat{\eta }\right)-2E\left(\left[\frac{A}{\hat{g}\left(L\right)}\left\{Y-\hat{Q}\left(L\right)\right\}+\hat{Q}\left(L\right)-m\left(Z\right)\right]\left\{\hat{m}\left(Z\right)-m\left(Z\right)\right\}\right)\;+E\left[{\left\{\hat{m}\left(Z\right)-m\left(Z\right)\right\}}^{2}\right] \end{array}$$

from which

$$\begin{array}{l} E\left[{\left\{\hat{m}\left(Z\right)-m\left(Z\right)\right\}}^{2}\right]=L\left(\hat{m},\hat{\eta }\right)-L\left(m,\hat{\eta }\right)\\ +2E\left(\left[\frac{A}{\hat{g}\left(L\right)}\left\{Y-\hat{Q}\left(L\right)\right\}+\hat{Q}\left(L\right)-m\left(Z\right)\right]\left\{\hat{m}\left(Z\right)-m\left(Z\right)\right\}\right) \end{array}$$

Here, the expectation in last second term can be further expanded as

$$\begin{array}{ll} & E\left([\frac{A}{g\left(L\right)}\left\{Y-Q\left(L\right)\right\}+Q\left(L\right)-m\left(Z\right)]\left\{\hat{m}\left(Z\right)-m\left(Z\right)\right\}\right)\;\;-E\left(\left[\frac{A}{g^{2}\left(L\right)}\left\{Y-Q\left(L\right)\right\}\right]\left\{\hat{g}\left(L\right)-g\left(L\right)\right\}\left\{\hat{m}\left(Z\right)-m\left(Z\right)\right\}\right)\;+E[\left\{1-\frac{A}{g\left(L\right)}\right\}\left\{\hat{Q}\left(L\right)-Q\left(L\right)\right\}\left\{\hat{m}\left(Z\right)-m\left(Z\right)\right\}]\;\;+E\left(\left[\frac{A}{{\bar{g}}^{3}\left(L\right)}\left\{Y-\bar{Q}\left(L\right)\right\}\right]{\left\{\hat{g}\left(L\right)-g\left(L\right)\right\}}^{2}\left\{\hat{m}\left(Z\right)-m\left(Z\right)\right\}\right)\;\;+E\left(\frac{A}{{\bar{g}}^{2}\left(L\right)}\left\{\hat{g}\left(L\right)-g\left(L\right)\right\}\left\{\hat{Q}\left(L\right)-Q\left(L\right)\right\}\left\{\hat{m}\left(Z\right)-m\left(Z\right)\right\}\right)\;=E\left(\left\{Q\left(L\right)-m\left(Z\right)\right\}\left\{\hat{m}\left(Z\right)-m\left(Z\right)\right\}\right)\;\;+E\left(\frac{g\left(L\right)}{{\bar{g}}^{3}\left(L\right)}\left\{Q\left(L\right)-\bar{Q}\left(L\right)\right\}{\left\{\hat{g}\left(L\right)-g\left(L\right)\right\}}^{2}\left\{\hat{m}\left(Z\right)-m\left(Z\right)\right\}\right)\;\;+E\left(\frac{g\left(L\right)}{{\bar{g}}^{2}\left(L\right)}\left\{\hat{g}\left(L\right)-g\left(L\right)\right\}\left\{\hat{Q}\left(L\right)-Q\left(L\right)\right\}\left\{\hat{m}\left(Z\right)-m(Z)\right\}\right). \end{array}$$

for $\bar{g}\left(L\right)=tg\left(L\right)+\left(1-t\right)\tilde{g}\left(L\right)$ for some *t* ∈ [0, 1] and $\tilde{g}\left(L\right)$ an element of a vector space large enough to contain $\hat{g}\left(L\right)$ and, likewise, $\bar{Q}\left(L\right)=tQ\left(L\right)+\left(1-t\right)\tilde{Q}\left(L\right)$ for $\tilde{Q}\left(L\right)$ an element of a vector space large enough to contain $\hat{Q}\left(L\right)$ . It follows that

$$\begin{array}{ll} E[{\left\{\hat{m}\left(Z\right)-m\left(Z\right)\right\}}^{2}] & =L\left(\hat{m},\hat{\eta }\right)-L\left(m,\hat{\eta }\right)\;+2E\left(\left\{Q\left(L\right)-m\left(Z\right)\right\}\left\{\hat{m}\left(Z\right)-m\left(Z\right)\right\}\right)\;+2E\left(\frac{g\left(L\right)}{{\bar{g}}^{3}\left(L\right)}\left\{Q\left(L\right)-\bar{Q}\left(L\right)\right\}{\left\{\hat{g}\left(L\right)-g\left(L\right)\right\}}^{2}\left\{\hat{m}\left(Z\right)-m\left(Z\right)\right\}\right)\;+2E\left(\frac{g\left(L\right)}{{\bar{g}}^{2}\left(L\right)}\left\{\hat{g}\left(L\right)-g\left(L\right)\right\}\left\{\hat{Q}\left(L\right)-Q\left(L\right)\right\}\left\{\hat{m}\left(Z\right)-m\left(Z\right)\right\}\right). \end{array}$$

By the Cauchy-Schwarz inequality, this can be upper bounded by

$$\begin{array}{ll} E\left[{\left\{\hat{m}\left(Z\right)-m\left(Z\right)\right\}}^{2}\right] & \leq L\left(\hat{m},\hat{\eta }\right)-L\left(m,\hat{\eta }\right)\;+2E\left(\left\{Q\left(L\right)-m\left(Z\right)\right\}\left\{\hat{m}\left(Z\right)-m\left(Z\right)\right\}\right)\;+2E{\left(\frac{g^{2}\left(L\right)}{{\bar{g}}^{6}\left(L\right)}{\left\{Q\left(L\right)-\bar{Q}\left(L\right)\right\}}^{2}{\left\{\hat{g}\left(L\right)-g\left(L\right)\right\}}^{4}\right)}^{1/2}\;E{\left[{\left\{\hat{m}\left(Z\right)-m\left(Z\right)\right\}}^{2}\right]}^{1/2}\;+2E{\left(\frac{g^{2}\left(L\right)}{{\bar{g}}^{4}\left(L\right)}{\left\{\hat{g}\left(L\right)-g\left(L\right)\right\}}^{2}{\left\{\hat{Q}\left(L\right)-Q\left(L\right)\right\}}^{2}\right)}^{1/2}E{\left[{\left\{\hat{m}\left(Z\right)-m\left(Z\right)\right\}}^{2}\right]}^{1/2}. \end{array}$$

Finally, it follows from the AM-GM inequality that for arbitrary *δ*_1_, *δ*_2_ > 0:

$$\begin{array}{ll} E\left[{\left\{\hat{m}\left(Z\right)-m\left(Z\right)\right\}}^{2}\right] & \leq L\left(\hat{m},\hat{\eta }\right)-L\left(m,\hat{\eta }\right)\;+2E\left(\left\{Q\left(L\right)-m\left(Z\right)\right\}\left\{\hat{m}\left(Z\right)-m\left(Z\right)\right\}\right)\;+\frac{1}{\delta_{1}}E\left(\frac{g^{2}\left(L\right)}{{\bar{g}}^{6}\left(L\right)}{\left\{Q\left(L\right)-\bar{Q}\left(L\right)\right\}}^{2}{\left\{\hat{g}\left(L\right)-g\left(L\right)\right\}}^{4}\right)\;+\frac{1}{\delta_{2}}E\left(\frac{g^{2}\left(L\right)}{{\bar{g}}^{4}\left(L\right)}{\left\{\hat{g}\left(L\right)-g\left(L\right)\right\}}^{2}{\left\{\hat{Q}\left(L\right)-Q\left(L\right)\right\}}^{2}\right)\;+\left(\delta_{1}+\delta_{2}\right)E\left\{{\left\{\hat{m}\left(Z\right)-m\left(Z\right)\right\}}^{2}\right\}, \end{array}$$

where the second term is non-negative by virtue of minimization (and exactly zero when the function class considered for *m*(.) contains $E\left\{Q\left(L\right)|Z\right\}$ ). We conclude that

$$\begin{array}{ll} E[{\left\{\hat{m}\left(Z\right)-m\left(Z\right)\right\}}^{2}] & \leq {\left(1-\delta_{1}-\delta_{2}\right)}^{-1}[\underset{\eta }{\sup}|L\left(m,\eta \right)-L\left(\hat{m},\eta \right)|\;+\frac{1}{\delta_{1}}E\left(\frac{g^{2}\left(L\right)}{{\bar{g}}^{6}\left(L\right)}{\left\{Q\left(L\right)-\bar{Q}\left(L\right)\right\}}^{2}{\left\{\hat{g}\left(L\right)-g\left(L\right)\right\}}^{4}\right)\;+\frac{1}{\delta_{2}}E(\frac{g^{2}\left(L\right)}{{\bar{g}}^{4}\left(L\right)}{\left\{\hat{g}\left(L\right)-g\left(L\right)\right\}}^{2}{\left\{\hat{Q}\left(L\right)-Q\left(L\right)\right\}}^{2})], \end{array}$$

where the error ${\left\{\hat{g}\left(L\right)-g\left(L\right)\right\}}^{4}$ and the product of the errors ${\left\{\hat{g}\left(L\right)-g\left(L\right)\right\}}^{2}$ and ${\left\{\hat{Q}\left(L\right)-Q\left(L\right)\right\}}^{2}$ in the last terms make the meta-learner less sensitive to slow convergence of the nuisance parameter estimators, provided that the positivity assumption holds (i.e., that *P*(*A* = 1|*L*) > 0 w.p.1).

## Appendix B

### Proof of Theorem 1

In this Appendix, we develop oracle bounds for the targeted meta-learner which minimizes the loss

$$L\left(m,\eta \right)\equiv E\left[A{\left\{Y-m\left(Z\right)\right\}}^{2}+\left(1-A\right){\left\{Q\left(L\right)-m\left(Z\right)\right\}}^{2}\right],$$

for *η* ≡ (*g*, *Q*). In particular, we will use that, by the targeting step, the above loss is sufficiently close to the orthogonal loss considered in Appendix A to deliver favourable oracle bounds.

A Taylor series expansion using directional derivatives shows that

$$\begin{array}{ll} L\left(\hat{m},\hat{\eta }\right) & =L\left(m,\hat{\eta }\right)-2E[\left\{AY+\left(1-A\right)\hat{Q}\left(L\right)-m\left(Z\right)\right\}\{\hat{m}\left(Z\right)-m\left(Z\right)\}]\;+E[{\left\{\hat{m}\left(Z\right)-m\left(Z\right)\right\}}^{2}]=L\left(m,\hat{\eta }\right)-2E\left([\frac{A}{\hat{g}\left(L\right)}\left\{Y-\hat{Q}\left(L\right)\right\}+\hat{Q}\left(L\right)-m\left(Z\right)]\left\{\hat{m}\left(Z\right)-m\left(Z\right)\right\}\right)+2E[\frac{A\{1-\hat{g}\left(L\right)\}}{\hat{g}\left(L\right)}\left\{Y-\hat{Q}\left(L\right)\right\}\left\{\hat{m}\left(Z\right)-m\left(Z\right)\right\}]\;+E\left[{\left\{\hat{m}\left(Z\right)-m\left(Z\right)\right\}}^{2}\right] \end{array}$$

from which

$$\begin{array}{ll} E\left[{\left\{\hat{m}\left(Z\right)-m\left(Z\right)\right\}}^{2}\right] & =L\left(\hat{m},\hat{\eta }\right)-L\left(m,\hat{\eta }\right)\;+2E\left([\frac{A}{\hat{g}\left(L\right)}\left\{Y-\hat{Q}\left(L\right)\right\}+\hat{Q}\left(L\right)-m\left(Z\right)]\left\{\hat{m}\left(Z\right)-m\left(Z\right)\right\}\right)\;-2E[\frac{A\{1-\hat{g}\left(L\right)\}}{\hat{g}\left(L\right)}\left\{Y-\hat{Q}\left(L\right)\right\}\left\{\hat{m}\left(Z\right)-m\left(Z\right)\right\}]. \end{array}$$

By the result of Appendix A (which resulted in identical expressions apart from the last term), it follows that

$$\begin{array}{ll} E\left[{\left\{\hat{m}\left(Z\right)-m\left(Z\right)\right\}}^{2}\right] & \leq {\left(1-\delta_{1}-\delta_{2}\right)}^{-1}\left[\underset{\eta }{\sup}|L\left(m,\eta \right)-L\left(\hat{m},\eta \right)|\right.\;\left.+\frac{1}{\delta_{1}}E\left(\frac{g^{2}\left(L\right)}{{\bar{g}}^{6}\left(L\right)}{\left\{Q\left(L\right)-\bar{Q}\left(L\right)\right\}}^{2}{\left\{\hat{g}\left(L\right)-g\left(L\right)\right\}}^{4}\right)\right.\;\left.+\frac{1}{\delta_{2}}E\left(\frac{g^{2}\left(L\right)}{{\bar{g}}^{4}\left(L\right)}{\left\{\hat{g}\left(L\right)-g\left(L\right)\right\}}^{2}{\left\{\hat{Q}\left(L\right)-Q\left(L\right)\right\}}^{2}\right)\right.-2E[\frac{A\{1-\hat{g}\left(L\right)\}}{\hat{g}\left(L\right)}\left\{Y-\hat{Q}\left(L\right)\right\}\{\hat{m}\left(Z\right)-m\left(Z\right)\}]]. \end{array}$$

To bound the last term in the above expression, we will first show that the expectation in that term is close to

$$\frac{1}{n}\overset{n}{\underset{i=1}{\sum }}[\frac{A_{i}\{1-\hat{g}\left(L_{i}\right)\}}{\hat{g}\left(L_{i}\right)}\left\{Y_{i}-\hat{Q}\left(L_{i}\right)\right\}\{\hat{m}\left(Z_{i}\right)-m\left(Z_{i}\right)\}],$$

which the targeting step in the proposed procedure directly aims to shrink. We will next study the order of magnitude of the latter term.

First, by Markov’s inequality, we have that the probability for

(11)
$$\begin{array}{ll} & \frac{1}{\sqrt{n}}\overset{n}{\underset{i=1}{\sum }}[\frac{A_{i}\{1-\hat{g}\left(L_{i}\right)\}}{\hat{g}\left(L_{i}\right)}\{Y_{i}-\hat{Q}\left(L_{i}\right)\}\{\hat{m}\left(Z_{i}\right)-m\left(Z_{i}\right)\}]\\ & -E[\frac{A\{1-\hat{g}\left(L\right)\}}{\hat{g}\left(L\right)}\{Y-\hat{Q}\left(L\right)\}\{\hat{m}\left(Z\right)-m\left(Z\right)\}] \end{array}$$

to exceed some constant *ϵ* > 0 in absolute value is upper bounded by

$$\frac{1}{\epsilon^{2}}E[\frac{A{\left\{1-\hat{g}\left(L\right)\right\}}^{2}}{{\hat{g}}^{2}\left(L\right)}{\left\{Y-\hat{Q}\left(L\right)\right\}}^{2}{\left\{\hat{m}\left(Z\right)-m\left(Z\right)\right\}}^{2}]\leq \frac{M^{2}}{\sigma^{2}\epsilon^{2}}E[{\left\{\hat{m}\left(Z\right)-m\left(Z\right)\right\}}^{2}]$$

if $|Y-\hat{Q}\left(L\right)|\leq M>0$ with probability 1, as we will assume; note that this is guaranteed to hold for dichotomous outcomes, whose analysis motivated this work. It thus follows that 1 over root-*n* times (11) is of the order $O_{p}\left(n^{-1/2}\right)E\left[{\left\{\hat{m}\left(Z\right)-m\left(Z\right)\right\}}^{2}\right]$ (and thus a lower order term).

### Proof of Proposition 1

Denote

$$C_{i}\equiv A_{i}\frac{1-\hat{g}\left(L_{i}\right)}{\hat{g}\left(L_{i}\right)}\left\{Y_{i}-\hat{Q}\left(L_{i}\right)\right\},$$

then it remains to study the order of magnitude of

$$\frac{1}{n}\overset{n}{\underset{i=1}{\sum }}C_{i}\left\{\hat{m}\left(Z_{i}\right)-m\left(Z_{i}\right)\right\}.$$

In the proposed procedure based on penalized sieve estimators, we restrict the optimization procedure to functions of the form $m\left(Z_{i}\right)=\sum_{j=1}^{\infty }\gamma_{j}b_{j}\left(Z_{i}\right)$ . Then

$$\frac{1}{n}\overset{n}{\underset{i=1}{\sum }}C_{i}\{\hat{m}\left(Z_{i}\right)-m\left(Z_{i}\right)\}=\frac{1}{n}\overset{n}{\underset{i=1}{\sum }}\overset{J_{n}}{\underset{j=1}{\sum }}C_{i}b_{j}\left(Z_{i}\right)\left({\hat{\gamma }}_{j}-\gamma_{j}\right)-\frac{1}{n}\overset{n}{\underset{i=1}{\sum }}\overset{\infty }{\underset{j=J_{n}+1}{\sum }}C_{i}b_{j}\left(Z_{i}\right)\gamma_{j},$$

since ${\hat{\gamma }}_{j}=0$ when *j* > *J*_*n*_. The penalization procedure used for the targeting step ensures (by Hölder’s inequality) that the first term

$$\left\|\frac{1}{n}\overset{n}{\underset{i=1}{\sum }}\overset{J_{n}}{\underset{j=1}{\sum }}\epsilon_{i}b_{j}\left(Z_{i}\right)\left({\hat{\gamma }}_{j}-\gamma_{j}\right)\right\|\leq {\left\|\frac{1}{n}\overset{n}{\underset{i=1}{\sum }}\overset{J_{n}}{\underset{j=1}{\sum }}\epsilon_{i}b_{j}\left(Z_{i}\right)\right\|}_{\infty }\|{\hat{\gamma }}_{j}-\gamma_{j}{\|}_{1}\leq \lambda \|{\hat{\gamma }}_{j}-\gamma_{j}{\|}_{1}$$

Here, the last inequality follows from the Karush-Kuhn-Tucker conditions, with *λ* the penalty term, which is of the order $O_{p}\left(\sqrt{\log J_{n}/n}\right)$ with *J*_*n*_ = *C*(*D*)*d* ^*D*′^ *n* ^1/3^ log^*D*′−1^(*n*) (see Theorem 5.1 in [25]) under the ellipsoid-type condition which restricts *m*(*Z*) to satisfy

$$\overset{\infty }{\underset{j=1}{\sum }}{\left(\frac{j}{\max\left(\log^{D-1}\;j,1\right)}\right)}^{2}\gamma_{j}^{2}\leq Q$$

for some constant *Q*. Further, from Corollary D.6 in [25] with *s* = 1 (in line with the above ellipsoid-type condition),

$$\|{\hat{\gamma }}_{j}-\gamma_{j}{\|}_{1}=O_{p}\left(\sqrt{\frac{\log J_{n}}{n}}n^{\frac{1}{3}}\;\log^{\frac{2\left(D-1\right)}{3}}\left(n\right)\right)$$

We conclude that the first term is

$$O_{p}\left(\log J_{n}n^{-2/3}\;\log^{\frac{2\left(D-1\right)}{3}}\left(n\right)\right)$$

Further, we have that

$$\frac{1}{n}\overset{n}{\underset{i=1}{\sum }}\overset{\infty }{\underset{j=J_{n}+1}{\sum }}C_{i}b_{j}\left(Z_{i}\right)\gamma_{j}=\frac{1}{n}\overset{n}{\underset{i=1}{\sum }}\epsilon_{i}\overset{\infty }{\underset{j=J_{n}+1}{\sum }}b_{j}\left(Z_{i}\right)\gamma_{j}+\frac{1}{n}\overset{n}{\underset{i=1}{\sum }}\overset{\infty }{\underset{j=J_{n}+1}{\sum }}C_{i}^{*}b_{j}\left(Z_{i}\right)\gamma_{j},$$

where

$$\epsilon_{i}\equiv A_{i}\frac{1-\hat{g}\left(L_{i}\right)}{\hat{g}\left(L_{i}\right)}\left\{Y_{i}-Q\left(L_{i}\right)\right\},$$

and

$$C_{i}^{*}\equiv A_{i}\frac{1-\hat{g}\left(L_{i}\right)}{\hat{g}\left(L_{i}\right)}\{Q\left(L_{i}\right)-\hat{Q}\left(L_{i}\right)\}.$$

Here, the first term has mean zero (namely, it has mean zero given *A* and *L* since $\hat{g}\left(L_{i}\right)$ is a functional of only the data in *A* and *L*) and variance

$$\begin{array}{ll} \frac{1}{n}E\left[\mathrm{Var}\left(\epsilon |Z\right){\left\{\overset{\infty }{\underset{j=J_{n}+1}{\sum }}b_{j}\left(Z_{i}\right)\gamma_{j}\right\}}^{2}\right] & \leq \frac{1}{n}\underset{z}{\sup}\mathrm{Var}\left(\epsilon |z\right)f_{Z}\left(z\right)\int {\left\{\overset{\infty }{\underset{j=J_{n}+1}{\sum }}b_{j}\left(z\right)\gamma_{j}\right\}}^{2}\;dz\\ & \leq \frac{1}{n}\underset{z}{\sup}\mathrm{Var}\left(\epsilon |z\right)f\left(z\right)\overset{\infty }{\underset{j=J_{n}+1}{\sum }}\gamma_{j}^{2}, \end{array}$$

where the last step follows from the chosen basis being orthonormal, and where we assume that the conditional variance Var(*ϵ*|.) and the density *f*_*Z*_(.) are bounded (which is a plausible assumption when there are no positivity violations). Further, by the approximation results for sieves in lemma C.5 and C.6 of [25] (see in particular the similar derivation leading to equation (60) in [25]), we have that $\sum_{j=J_{n}+1}^{\infty }\gamma_{j}^{2}$ is of the order ${\left(\log^{D-1}\;n/n\right)}^{\frac{2}{3}}$ (or less). We conclude (by calling on Markov’s inequality) that the first term is $O_{p}\left(n^{-1/2}{\left(\log^{D-1}\;n/n\right)}^{\frac{1}{3}}\right)=O_{p}\left(n^{-5/6}\;\log^{\left(D-1\right)/3}\;n\right)$ .

We next bound the second term. First, note that, by a similar reasoning as in the previous paragraph, its variance is readily shown to be upper bounded by a term of the order $n^{-1}{\left(\log^{D-1}\;n/n\right)}^{\frac{2}{3}}$ . We therefore conclude that the second term equals

$$E\left\{C^{*}\overset{\infty }{\underset{j=J_{n}+1}{\sum }}b_{j}\left(Z\right)\gamma_{j}\right\}+O_{p}\left(n^{-5/6}\;\log^{\left(D-1\right)/3}\;n\right)$$

Using the Cauchy-Schwarz inequality, we further have that

$$\left\|E\left\{C^{*}\overset{\infty }{\underset{j=J_{n}+1}{\sum }}b_{j}\left(Z\right)\gamma_{j}\right\}\right\|\leq E{\left(C^{*2}\right)}^{1/2}\;E{\left\{{\left(\overset{\infty }{\underset{j=J_{n}+1}{\sum }}b_{j}\left(Z\right)\gamma_{j}\right)}^{2}\right\}}^{1/2}.$$

By a similar reasoning as in the previous paragraph,

$$E\left\{{\left(\overset{\infty }{\underset{j=J_{n}+1}{\sum }}b_{j}\left(Z\right)\gamma_{j}\right)}^{2}\right\}$$

is readily shown to be upper bounded by a term of the order ${\left(\log^{D-1}\;n/n\right)}^{\frac{2}{3}}$ . We conclude that

$$E\left\{C^{*}\overset{\infty }{\underset{j=J_{n}+1}{\sum }}b_{j}\left(Z\right)\gamma_{j}\right\}$$

is of the order *O*(*n* ^−*b*−1/3^ log^(*D*−1)/3^ *n*) for some *b* ≥ 1/2.

Putting it all together, we conclude that

$$\begin{array}{ll} \frac{1}{n}\overset{n}{\underset{i=1}{\sum }}\overset{\infty }{\underset{j=J_{n}+1}{\sum }}C_{i}b_{j}\left(Z_{i}\right)\gamma_{j} & =O_{p}\left(\log J_{n}n^{-2/3}\;\log^{\frac{2\left(D-1\right)}{3}}\;n\right)+O_{p}\left(n^{-5/6}\;\log^{\left(D-1\right)/3}\;n\right)\\ & \;+O_{p}\left(n^{-b-1/3}\;\log^{\left(D-1\right)/3}\;n\right). \end{array}$$

## Appendix C

See Figures 4–8.
